# Optimizing experimental conditions: the role of buffered environments in microbial isolation, physiological studies, and taxonomic characterization

**DOI:** 10.1128/aem.01728-24

**Published:** 2025-05-14

**Authors:** Om Prakash, Ujjwala Waghmare, Ashvini Chauhan, Yogesh Patil

**Affiliations:** 1 Symbiosis Centre for Climate Change and Sustainability, Symbiosis International (Deemed University) 642527 , Lavale, India; 2 Environmental Biotechnology School of the Environment, Florida A&M University 7822, Tallahassee, Florida, USA; 3 Symbiosis Centre for Research and Innovation, Symbiosis International (Deemed University) 642527 , Lavale, India; Washington University in St. Louis, St. Louis, Missouri, USA

**Keywords:** pH range and optima, organic buffers, novel bacterial taxa

## Abstract

Using a buffered medium is considered essential for enriching and cultivating novel microbial taxa, studying their pH range and optima, and conducting different physiological experiments. Experimental evidence showed that different buffer compounds impact microbial physiology and cell growth differently, and some of them exert toxic and inhibitory effects on organisms under study. Laboratory growth media supplemented with incompatible buffers could also suppress the organism’s growth. Therefore, the selection of buffers without the knowledge of their implications on cell growth and physiology in such experiments yields an inaccurate estimate of their physiological abilities and pH range and optima. In this paper, the authors argue against the use of buffered medium to enrich and isolate novel taxa and suggest determining the pH range and optima using unbuffered medium for taxonomic description and physiological characterization. Based on previous literature and our observations, we recommend the use of rich universal laboratory growth medium with their pH adjusted using 1 N NaOH and/or 1 N HCl for such studies, except in cases where the organism cannot grow in such media. However, the pH of the growth medium must be continuously monitored, and in cases where the medium’s buffering capacity is compromised, a suitable pH buffer with only a neutral effect on cell growth must be used for more accurate physiological experiments with that organism in the future. Based on the inhibitory effects of buffers on different cells (prokaryotes and eukaryotes) and physiological activities, in this manuscript, we also recommend that the compatibility of the buffers should be first screened before starting any physiological experiments, and any buffer compound should be avoided in the culture medium during the designing of the culturomics protocols for the cultivation of novel taxa from natural samples.

## INTRODUCTION

An ecosystem is a community of living organisms that coexist and interact with each other, as well as with their surrounding physical environment. Earth’s ecosystem is generally classified as terrestrial and aquatic; the terrestrial ecosystem constitutes grassland, forest, desert, tundra, and mountains, while aquatic ecosystems contain freshwater and marine habitats. Any ecosystem’s buffering capacity (capacity to resist pH change) is important in ecosystem structure and functions. Every ecosystem has its own natural buffer capacity, and a specific class of buffer compounds maintains the pH range of different ecosystems (1–4). For example, bicarbonate buffers maintain the buffering capacity of the ocean and blood, while a phosphate buffer system generally maintains the internal environment of all cells. An ecosystem’s buffering capacity is critical for maintaining vegetation and community structure of microeukaryotes and prokaryotes. Contamination of the ecosystem (due to sudden entry of carbon, nitrogen, and chemicals), climate change, and external amendments in agricultural soil change the buffering capacity of the ecosystem (5, 6), consequently changing the ecosystem’s pH. For example, it has been extensively reported that certain agricultural practices and amendments lead to changes in the soil pH (7–12), which leads to changes in the structure and functions of the soil microbial communities.

Microbes are critical components of any ecosystem and leading providers of ecosystem services, including pollutants degradation, wastewater treatment, plant growth promoters, waste degradation and management, greenhouse gas emission, global warming and climate change, carbon sequestration and climate risk mitigation, and biogeochemical cycling of materials (13–16). In addition, microbes are the backbone of every ecological system and play a critical role in public and environmental health. To understand the roles of microbes in ecosystem services, it is necessary to explore their potential in biotechnological growth, agricultural practices, therapeutic approaches, climate adaptation, and mitigation (14, 15). Soil is the most explored ecosystem in microbial cultivation, but despite all the efforts, only 1–10% of soil microbiota has been cultured (17). Unlike soil, all other terrestrial and aquatic ecosystems are less explored regarding the cultivation of novel microbial taxa. The buffering capacity of any ecosystem plays a critical role in maintaining the structures and functions of microorganisms in a steady state. Any change or dysbiosis in buffering capacity leads to a pH shift, which results in microbial extinction, community shift, altered ecosystem functionality, and biogeochemical cycling of materials. Knowledge of natural buffers also plays a vital role in cultivating novel taxa, understanding their physiological potentials/functionality in natural ecosystems, and choosing appropriate buffer systems to explore their physiological potential. This paper presents some novel insights into the following aspects:

1. The natural buffering capacity of ecosystems.
2. Why selecting appropriate buffers is essential for physiological experiments and the cultivation of novel taxa?
3. Why is every buffer not suitable for every type of microbe?
4. What inhibitory effects are exerted by inorganic buffers on biological systems, and why are biological buffers suitable for physiological experiments?

## IS IT ALWAYS RIGHT TO USE BUFFERED CONDITIONS FOR CULTIVATION AND PHYSIOLOGICAL STUDIES?

The study of physiological features of bacteria, such as the range and optima for pH, salinity, and temperature, under similar laboratory conditions, is a common practice for the characterization of novel taxa and, essentially so, for its description in the International Journal of Systematic and Evolutionary Microbiology, Systematic and Applied Microbiology, and many other microbiological journals dealing with cultivation and taxonomy. Bacteria generally produce organic acids or alkali (ammonia) during their growth, and gradual accumulation of organic acids or alkali challenges the buffering capacity of the growth medium, sometimes eventually leading to a change in its pH. Hence, using pH buffers that resist this change in the pH of the growth medium is recommended during such physiological experiments. Due to this fact, while reviewing descriptions of novel taxa, most reviewers insist that authors use pH buffers to assess the pH range and optima. However, due to a lack of in-depth knowledge about cellular toxicity, cell compatibility, and inhibitory effects of buffers on microbial growth, many bacterial taxonomists are not careful and tend to select any buffer compounds (inorganic/organic) merely to satisfy the reviewers' comments regarding using buffers during pH experiments.

Consequently, it diminishes the chances of publishing a biologically accurate estimate of the pH range and optima of novel taxa. Therefore, it is only seldom appropriate to use pH buffer while cultivating and investigating the pH range and optima of bacteria without understanding the compatibility and toxicity of selected buffers on microbial cells.

## WHY MIGHT BUFFERS NOT BE MANDATORY DURING ENRICHMENT, CULTIVATION, AND INITIAL CHARACTERIZATION?

From a biodiversity perspective, the authors argue that using a pH buffer might not be necessary for the initial screening of the organisms to study pH range and optima for characterizing the novel taxa and for discovering new microbes from any natural ecosystem. The scientific explanations for this context are provided in the subsequent paragraphs.

First, there is not a single buffer that works in all physiological pH ranges, and it becomes essential to use different buffers (with different p*K*a values) to study the pH range and optima of any novel taxa (https://support.nanotempertech.com/hc/en-us/articles/23850406317713-Buffer-Considerations). However, each buffer would have a variable effect on the growing organism throughout the pH range. Hence, cell permeability, solubility, ionic strength, inertness, and complex-forming capacity of the buffer compounds with media components should be studied before the selection of the buffer to maintain the pH in a physiological experiment. For instance, while TES buffer (*N*-[Tris(hydroxymethyl)methyl]-2-aminoethanesulfonic acid; p*K*a = 7.4) works between pH 6.8 and 8.2, MES (2-(*N*-Morpholino)-ethanesulfonic acid; p*K*a = 6.10) works best between pH 5.5 and 6.7, whereas citrate buffers only provide good buffering between pH 3.0 and 6.2 (18). In contrast to these organic buffers, inorganic buffers are unsuitable for biological systems as they are reactive and affect their growth and activities. Some buffer compounds, such as Tris (tris(hydroxymethyl)aminomethane), permeate into the cell cytoplasm and disturb the natural buffering capacity of the cell, consequently inhibiting growth or killing the cells (19). In addition, phosphate buffers provide more ionic strength than zwitterionic biological buffers to achieve the same pH (pH 7.5) (20).

Further, varied concentrations of these different buffers are used to achieve the appropriate buffering capacity. If one uses 50 mM of TES to achieve the buffering conditions in the pH range 7–8, then it is rationally correct to use 50 mM of MES as well to achieve buffering in the pH range 5–6. However, this is not the case. Using multiple buffers to maintain different pH conditions in the growth medium results in a non-homogenous chemical environment concerning its composition and concentration while determining the organisms' pH range and optima. Because different buffers affect the bacterium differently (stimulatory, inhibitory, or lethal) and consequently its growth and optical density of the medium, such assessments are likely to be biased.

It has also been found that some buffering compounds exert inhibitory effects on bacterial growth when used in a culture medium to maintain the buffering capacity, and the same bacterium grew better when the pH of the medium was simply adjusted using 1 N NaOH and 1 N HCl without buffer. For example, some *Rhodanobacter* strains showed little or no growth at pH 5 when HOMOPIPES (homopiperazine-*N*, *N*-bis-2-(ethanesulfonic acid)) was used as buffer (21). In contrast, their growth was optimal at pH 4 and below, when the medium pH was simply adjusted using 1 N HCl (personal observation). Similarly, some alkaliphilic bacteria isolated from dairy effluents grew at pH 10 when adjusted using 1 N NaOH, while some of them were inhibited when glycine NaOH buffer was used (22). Inhibition of bacterial growth by buffer compounds during the optimization of the medium has also been reported during some physiological experiments mentioned below. Hence, it is imperative that in such cases, the organism must first be well characterized for its sensitivity to different constituting compounds of the buffer. For the description of novel taxa, which is primarily a taxonomic study, performing such a thorough investigation is highly unlikely. While one may argue that understanding the physiological capacity of an organism is an integral part of polyphasic taxonomy, it is also logical to understand that a criterion that varies with the composition of the growth media is atypical of such an approach.

Second, the pH of an unbuffered growth medium does not change suddenly after inoculation of the organism (23). It can only change upon the accumulation of sufficient organic acids or alkalis as a consequence of bacterial growth (not always, because not just acids or alkali production but other factors such as CO_2_ concentration in water, temperature, carbonate and bicarbonate concentrations, and organic materials are also responsible for the change in pH of an un-buffered medium) (24). However, this change in pH does not always happen because the generation of organic acid (carboxylic acid) and ammonia in the medium neutralizes the effect (25). Furthermore, the accumulation of organic acid or ammonia will also depend upon the amount of carbon or nitrogen subjected in the medium. A high C/N ratio will lead to a high amount of organic acid formation, thus acidifying the medium, while a high amount of nitrogen (low C/N ratio) might lead to high ammonia formation, thus making the medium alkaline. Therefore, the C/N ratio is the key parameter and needs optimization, especially when the inorganic medium is being used. Given the number of cells in the inoculum, this suggests that bacterial growth has reached high enough density to cause such an effect, and throughout this duration, the organism was able to grow without much change in the pH of the growth medium (no need for buffer) (26). Therefore, by optimizing those factors, the initial stage of the medium (medium after inoculation till sufficient growth) may not require any buffers to resist changes in pH due to low numbers of cells. A chemostat is a special fermenter that operates on the concept of continuous culture with minimal change in the chemical composition of the medium. Several factors play a role in pH change during the cultivation of cells in chemostat for physiological studies, including the rate of supply of fresh medium and removal of spent medium with waste and by-products, nature of the strain(s) (acid/alkali producer) under cultivation, medium composition, and culture conditions. Maintaining the steady state in a chemostat is one of the ways to minimize pH change in an unbuffered medium to study the impact of buffer on growth and cell physiology. The use of an unbuffered medium with minimum sugar and nitrogen to prevent excess acid and ammonia release is also a good way to control pH in an unbuffered medium (27). It is also advisable here that running the control trials to set the steady state in growth without pH change before the experiment is a must to decide the possibility of running cultivations without buffers.

Rationally, most organisms live in an open system with continuous changes in conditions. Different pH conditions are known to activate certain pathways in bacteria, such as the *Bar*A (bacterial adaptive response)/*Uvr*Y two-component system in *Escherichia coli* (28). It is worth noting that although the pH of the unbuffered LB medium changed by almost 3.5 units in 400 min, the strain adapted to these changing conditions and grew as well as the buffered medium at pH 5 (28). This suggests that organisms have a mechanism to adapt to the changing pH conditions in the growth medium. It has been observed that bacteria can adapt to pH shifts, and every species has its pH range and optimum for growth. It indicates that the adaptation for pH shift can occur within certain limits of pH shifts. However, if the pH change exceeds their adaptive capacity or range (in terms of magnitude or duration), microbes might not maintain their survival, and their population can decline. Thus, their adaptability to pH change is limited depending on species and environmental factors. That is one of the reasons behind the loss of microbial diversity in environmental systems where the buffering capacity is altered (Fig. 2). In contrast, while using a buffered medium allows the organism to grow, it could also force it to cope with the stressful conditions created by the buffer compound used in a medium for resisting the change in pH by neutralizing one of the end products during its growth. It is very likely that this now “neutralized” end product could have otherwise served as a precursor of an essential compound required for the organism’s growth. In such a case, one is not getting a true representation of the “optimal” growth of the organism. Hence, for mere taxonomic purposes, we believe that buffered conditions should not be essential.

Third, synthetic or minimal media that lack sufficient organic load are typically more prone to fluctuations in pH, and the use of buffers in such media is justified. In contrast, most physiological experiments to determine the pH range and optima of bacteria are carried out in rich organic media, such as Luria broth, trypticase soy broth, and nutrient broth, which provide natural buffering capacity to the medium and experience the least pH fluctuation. Compounds like peptone and extracts from beef, meat, yeast, and soybean meal, which are constituents of rich media, impart natural buffering capacity to the medium (29, 30). Additionally, some such components protect the growing organisms from pH stress under certain conditions (31). For instance, yeast extract protects bacteria under acidic conditions (32). Thus, these universal laboratory media with their pH adjusted using 1 N NaOH and 1 N HCL can provide the organism with a more homogenous chemical environment. The use of such universal laboratory media that provide unbiased data for pH range and optima should be encouraged to describe novel taxa.

## OPTIMIZATION OF BUFFERS IS A MUST FOR AN ACCURATE REPRESENTATION OF PHYSIOLOGICAL POTENTIAL

Due to ease in cultivation and characterization, almost all culture collections are dominated by copiotrophs, and representation of other groups like oligotrophs and extremophiles is lesser due to their difficult cultivation and preservation strategies (33). The concept of buffer compatibility discussed in the scientific literature is mainly based on the behaviors of previously isolated copiotrophs which generally survive in a wider pH range (33). Furthermore, organisms like marine neutrophils, acidophiles, and alkaliphiles isolated from natural buffer systems like seawater (CO_2_ bicarbonate buffer system), acid mine drainage (mineral acidity and CO_2_ buffer system), and alkaline habitats (CO_2_-bicarbonate-carbonate buffers) show survival in a narrow range of pH (34), but the study of pH range and optima using neutral and suitable organic buffers is still required to better understand the physiology and functionality of the organisms. In addition, oligotrophic media experience more fluctuations in pH and provide low protection to the cells due to low organic load. Therefore, selecting the right buffers for the study of oligotrophs is mandatory to prevent fluctuations in pH. Only limited studies are available on the inhibitory or lethal effects of buffer compounds on the cell structure and physiological behavior of the organisms because of the researchers' pre-existing mindset that using buffers is safe and improves the authenticity of the experiments. Due to the time-consuming nature of physiological experiments, only a few researchers try to take the pain of studying the optimization of buffers in terms of cellular compatibility with organisms under investigation. It is essential to understand what buffer compounds and in what concentration are neutral with organisms under investigation and do not alter the physiological behavior of organisms or least affect the cellular and physiological characteristics. Often, we neglect the fact that buffers can change the physiological behaviors of organisms and use the buffer recommended by others mindlessly in our experiments. Every buffer has different effects on different organisms. It can slow down the growth rate, suppress or stimulate the physiological traits of organisms, or even show lethal or inhibitory effects on cells in higher concentrations.

## EXAMPLES OF INHIBITORY EFFECTS OF BUFFERS ON MICROBIAL CELLS AND PHYSIOLOGICAL TRAITS

**Fig 1:**
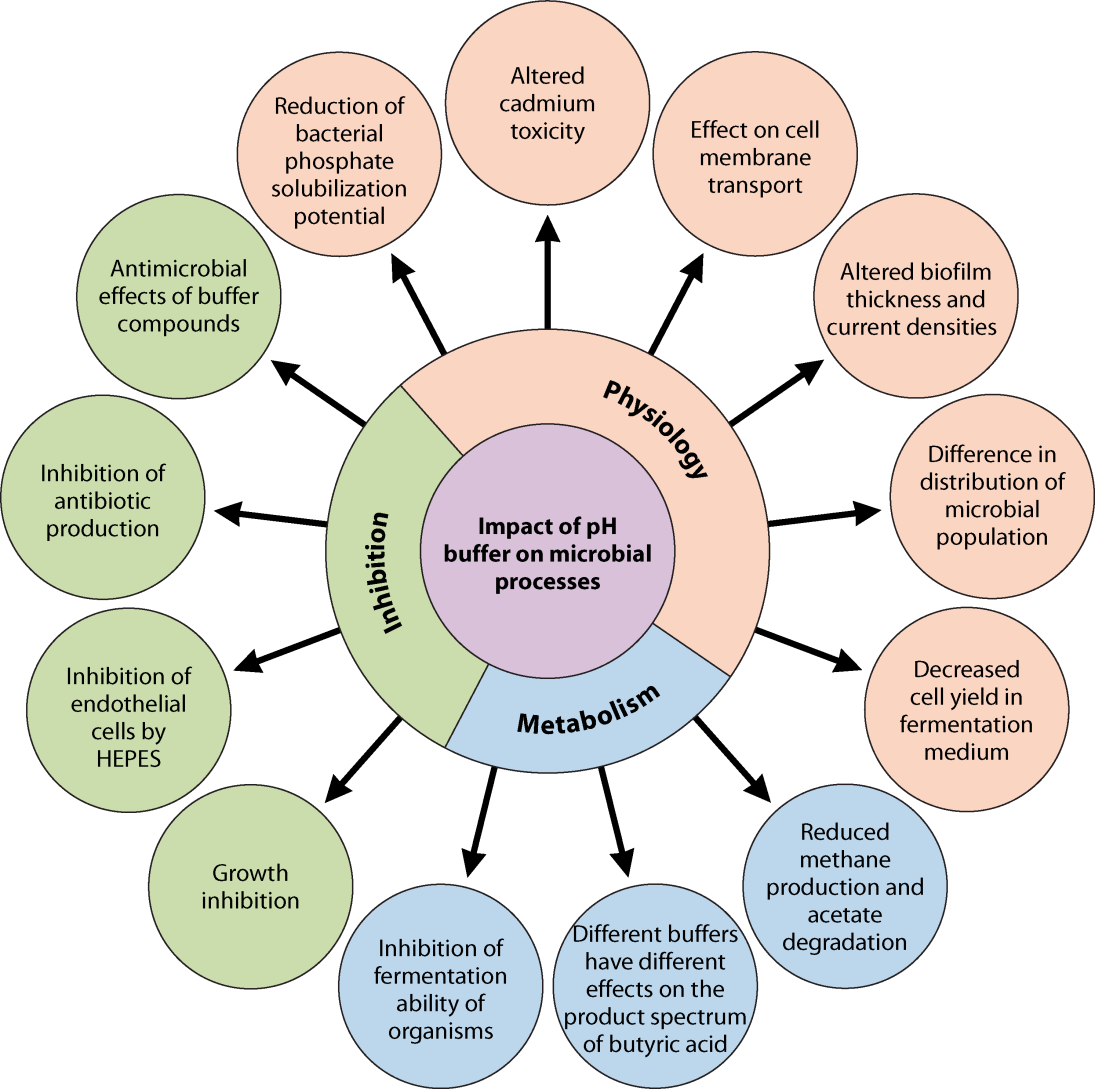
Diagram represents the impact of different buffer compounds on the inhibition of various metabolic and physiological processes in different microbial taxa. The figure is conceptualized from published data for which appropriate references are cited in Tables 1 and 2.

Several examples from past studies about the inhibitory effects of various buffers on different types of microbial cells (both prokaryotes and eukaryotes) are presented in Tables 1 and 2. Past studies on this line indicate that the compatibility of the used buffer compounds is not universal with all types of cells, and their behavior varies with the various organisms (Tables 1 and 2). It has been proven that buffer compounds can modify molecular and macroscopic properties of cells by altering the conformational changes and electrostatic stability in proteins and affecting the physiological behaviors of the cells (35–39). A survey on the impact of different buffer compounds on various cellular activities, including growth inhibition, cellular toxicity, the toxicity of heavy metals, and physiological traits, is presented in Table 1. Data on the adverse effects of buffers on the physiological activities of cells revealed that these buffers mediated molecular structure as well as macroscopic properties of the cell, which affect the physical activities of prokaryotes like reduction in phosphate solubility (40), altered cadmium toxicity for bacteria (41), methane production and acetate degradation (42), growth inhibition (30, 31), cell membrane transport, inhibition of antibiotic production (43), altered biofilm thickness and current densities (44), differences in the distribution of microbial population (45), inhibition of growth of *Staphylococcus aureus* in the presence of phosphate buffer (46), inhibition of fermenting ability of ethanol-producing organisms (30, 47, 48), decrease in cell yield of fermentation medium (49) and antimicrobial effects of different buffer compounds (50). The adverse effects of buffer components on eukaryotic cells include toxic effects on plant tissue culture (31), deleterious effect of TRIS on growth and pigment production of *Gracilaria birdiae* (34), and inhibition of endothelial cells by HEPES (35). Almost all buffering substances have physiological effects of their own (51). Figure 1 presents data on the impacts of different buffer compounds on the inhibition of various metabolic and physiological processes in different microbial taxa.

**TABLE 1:** Studies on the physiological effects of buffers on prokaryotes

S no.	Study	Inference	Reference
1.	Selection of pH buffers for use in conductimetric microbiological assays.	Buffers are the major source of conductivity changes in culture media.	(52)
2.	Sources of conductance changes during bacterial reduction of trimethylamine oxide to trimethylammonium in phosphate buffer.	The conductivity changes in *E. coli* are influenced by the presence of phosphate buffer.	(53)
3.	Effect of temperature, pH, and buffer presence on ethanol production from synthesis gas by *“Clostridium ragsdalei.”.*	In syngas fermentation, *Clostridium ragsdalei* shows a high ethanol production rate in the media lacking buffer.	(47)
4.	Effect of buffered acidulant systems on the survival of some food poisoning bacteria in medium acid media.	The buffered food acidulants are more bactericidal than unbuffered when studied against *Salmonella blockley, E. coli, Staphylococcus aureus*, and more bacteriostatic against *Streptococcus faecalis*.	(54)
5.	Toward the effect of buffer system composition in dehydrogenase activity of *Escherichia coli* and its electrochemical activity in mediated bioanode.	The composition of the buffer affects the viability and dehydrogenase activity of *E. coli*.	(55)
6.	Buffering reduces phosphate solubilizing ability of selected strains of bacteria.	The bacterial efficiency of phosphate solubilization decreases in buffered media compared to non-buffered media.	(40)
7.	The effect of pH and potassium phosphate buffer on the toxicity of cadmium for bacteria.	An increase in the pH of plate count agar led to increased toxicity of cadmium for *Micrococcus luteus, Staphylococcus aureus, Clostridium perfringens, Pseudomonas,* and *E. coli*.	(41)
8.	Influence of bicarbonate buffer on the methanogenetic pathway during thermophilic anaerobic digestion.	A high level of bicarbonate buffer reduces methane production and acetate degradation rates.	(42)
9.	Influence of inorganic phosphate and organic buffers on cephalosporin production by *Streptomyces clavuligerus*.	High concentrations of potassium phosphate and tris buffer inhibit antibiotic production in *Streptomyces clavuligerus*.	(43)
10.	Antimicrobial effect of citrate buffer with antibiotic.	Amoxiclav combined with citrate buffer shows antimicrobial effects on standard strains of microorganisms. (*C. albicans* ATCC10231*, E. coli* ATCC25922*, S. aureus* ATCC25923, *E. faecalis* ATCC29213, *M. luteus* ATCC4698, *S. epidermidis* ATCC14990).	(50)
11.	Effect of buffering on the phosphate-solubilizing ability of microorganisms.	The phosphate-solubilizing microbes were able to solubilize rock phosphate in unbuffered media but failed to solubilize it in buffered media.	(56)
12.	The effect of pH and buffer concentration on anode biofilms of *Thermincola ferriacetica*.	High concentration of bicarbonate buffer results in large biofilm thickness, whereas a lower concentration produces higher current densities.	(44)
13.	Effect of pH and buffer on butyric acid production and microbial community characteristics in bioconversion of rice straw with undefined mixed culture.	The difference in pH and buffers influencing the distribution of microbial populations. Also, different buffers have different effects on product spectrum in butyric acid production.	(57)
14.	Effect of phosphate buffer on *Staphylococcus aureus* growth at a reduced water activity.	In the presence of phosphate buffer, the inhibition of growth of *Staphylococcus aureus* was observed.	(46)
15.	Influences of buffer systems on enzymatic saccharification of rice husk holocellulose and fermenting ability of various ethanol-producing microorganisms.	Higher buffer concentrations of citrate and acetate buffer were found to inhibit the fermenting ability of ethanol-producing organisms.	(48)
16.	Effects of organic buffers on batch fermentations of *Zymomonas mobilis* in a synthetic and complex medium.	Acetic acid/acetate buffer increases the specific productivity but decreases the cell yield of *Zymomonas mobilis* in fermentation medium.	(49)
17.	A hidden pitfall in the preparation of agar media undermines microorganism cultivability.	Reactive oxygen species produced when phosphate and agar are autoclaved together can inhibit the growth of certain microbial taxa.	(58)

**TABLE 2:** Studies on the physiological effects of buffers on eukaryotes

S no.	Study	Inference	Reference
1.	Not only pH. Specific buffer effects in biological systems.	Buffers modify molecular and macroscopic properties of cells.	(38)
2.	Role of buffers in protein formulations.	Buffers can alter the colloidal stability of proteins, modulate interfacial damage, and can lead to the destabilization of proteins.	(39)
3.	Buffer-specific effects arise from ionic dispersion forces.	The zeta potential of lysozyme varies significantly in tris, phosphate, and citrate buffers.	(59)
4.	The impact of pH on catalytically critical protein conformational changes: the case of the urease, a nickel enzyme.	pH modulates the conformation of the mobile flap (structural motif) of urease enzyme.	(36)
5.	Effect of buffer on protein stability in aqueous solutions: A simple protein aggregation model.	Buffer molecules adsorb on the protein surface and modulate their electrostatic stability.	(35)
6.	Use of organic buffers in plant tissue-culture systems.	Tris buffer was toxic on tested plant tissue culture systems in all concentrations, and MES was toxic when used in high concentrations.	(60)
7.	The adverse effects of HEPES, TES, and BES zwitterion buffers on the ultrastructure of cultured chick embryo epiphyseal chondrocytes.	HEPES, TES, and BES buffers affect cell membrane transport and secretion systems in chick embryos, leading to vacuole accumulation and discharge of membrane-bound vacuoles into the extracellular matrix.	(37)
8.	Deleterious effect of TRIS buffer on growth rates and pigment content of *Gracilaria birdiae*.	The higher growth rates of *Gracilaria birdiae* were observed in PES without TRIS when compared to PES with TRIS.	(61)
9.	HEPES may stimulate cultured endothelial cells to make growth-retarding oxygen metabolites.	HEPES stimulates bovine pulmonary artery endothelial cells to make toxic oxygen metabolites that contribute to decreased cell growth.	(62)
10.	Analysis of pH and buffer effects on flucytosine activity in broth dilution susceptibility testing of *Candida albicans* in two synthetic media.	The growth rate of *Candida albicans* in yeast nitrogen base (YNB) and synthetic amino acid medium-fungal (SAAMF) was reduced when pH was raised by adding buffer.	(63)
11.	Curtailing citrate buffer inhibition effect on *S. cerevisiae* to enhance the fermentability of cellulosic hydrolysate.	Metabolic growth of *S. cerevisiae* reduces in the presence of a high concentration of citrate buffer.	(64)

It is generally considered that unlike inorganic buffers, Good buffers, or organic buffers, are better and safe for biological systems and are recommended for physiological experiments (65). Still, past data indicate that even organic buffers like MES, HEPES, TES, and BES can change the physiological traits of cells (37, 62).

Thus, after evaluation of experimentation-based studies on the impacts of buffer compounds (used for pH adjustment) on the growth and physiological activities of different classes of microorganisms, it is clear that not all the buffer compounds are inert, and some of them can exert positive or negative impacts on growth and physiological activities of microorganisms. Generally, organic buffers or Good buffers are considered safe, but some of them also interact with microorganisms and inhibit or stimulate certain processes. The impact depends on the type of microorganisms, their cellular structure, and physiological processes. The buffer compounds used for pH adjustment can hamper growth and physiological activities (Tables 1 and 2). However, due to the limited or moderate impact and inhibition of buffers on the physiological activities of cells, less attention has been given to this aspect. Most of the researchers neglected the buffer’s impact and considered that it is the organism’s inability to grow or perform the activities. Consequently, only limited research has been done, and a small amount of literature is available on this aspect. In order to select the right buffer for different physiological experiments, extensive future experiments using different classes of microbes are imperative. A database of the impact of different buffer compounds on growth and various physiological activities is essential. It not only assists in reducing the physiological stress of cells during experimentation but also reduces cultivation costs and improves yields and recovery of taxa during isolation.

## ALTERED NATURAL BUFFERING, ACIDIFICATION, CULTUROMICS, AND MICROBIOLOGICAL PROCESSES

**Fig 2:**
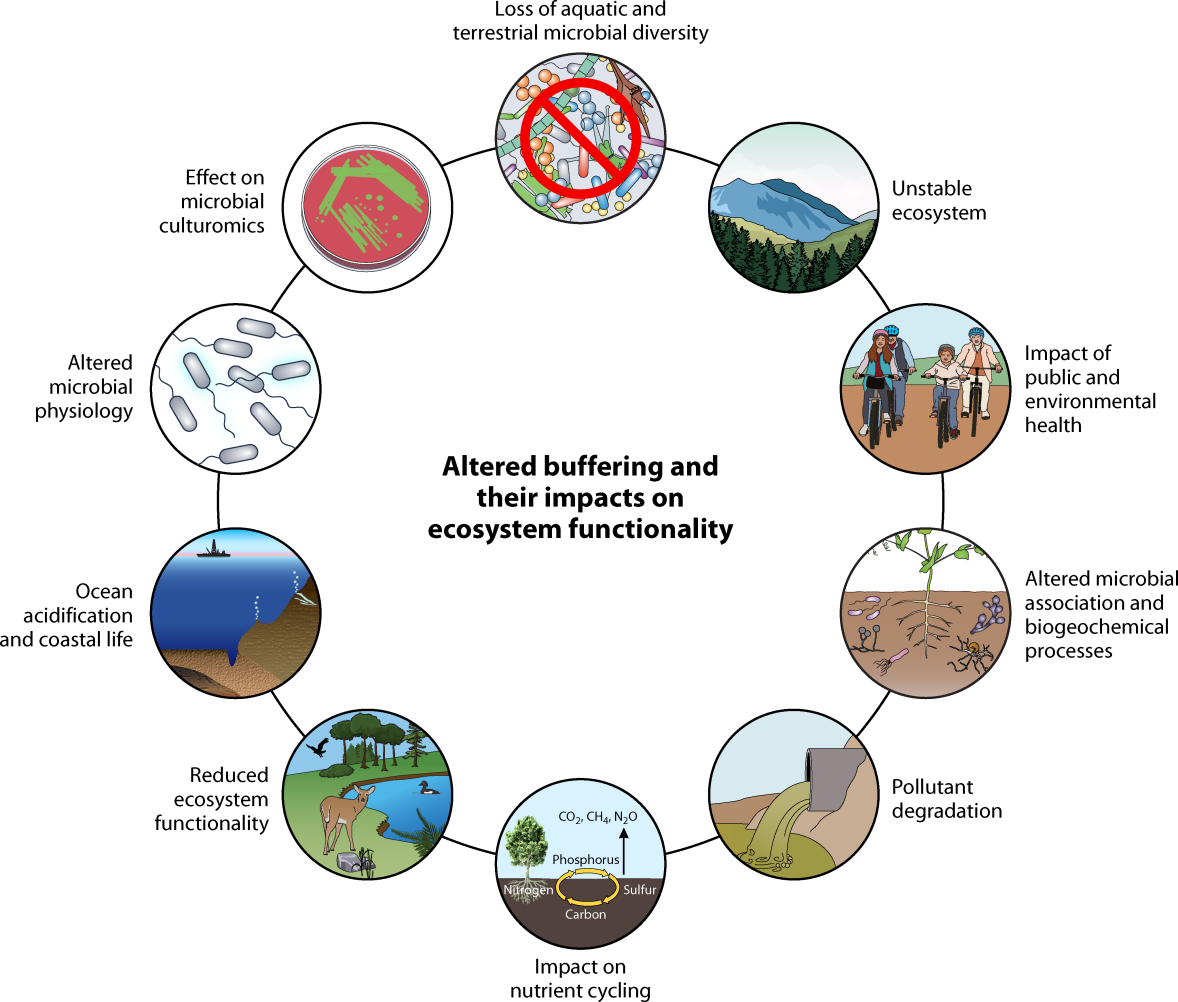
The diagram illustrates one example of the correlations of altered natural buffering of the ecosystems and its impact on biodiversity loss, ecosystem functionality, and environmental health.

Every ecosystem (aquatic and soil) has its natural buffering capacity, which nullifies the impact of sudden entry of acid or base and resists the change in the ecosystem functionality. Increasing atmospheric CO_2_, acidic/alkaline industrial wastewater, acid rains, and so on, can alter the natural buffering capacity and impact the microbial community structure and functions. Figure 2 represents the correlations of altered natural buffering of the ecosystems and its impact on biodiversity loss, ecosystem functionality, and environmental health. Even milder changes in ecosystem pH can significantly impact community structure and its associated functionality. Ocean acidification is one of the best examples of reducing ecosystem buffering and its impact on ecosystem functionality. The recent data from the Keeling curve indicates that environmental CO_2_ concentration has been increasing substantially to 408 ppm in 2015 from 315 ppm in 1958 due to excess anthropogenic carbon emissions in the environment (66). Oceans are one of the significant sinks of CO_2_. The buffering capacity of oceans depends on atmospheric CO_2_ concentration (67). Atmospheric CO_2_ dissolves in the ocean and forms carbonic acid, which further dissociates into HCO_3_^−^ and CO^2−^_3_. In addition to atmospheric CO_2_, CO_2_ released during the metabolic activities of inhabitants and the collision of underground tectonic plates is also absorbed in the ocean and contributes to the ocean acidification process (68). Due to the reversible nature of the reaction, they nullify the impact of the influx of acid and base and maintain the buffering capacity. However, due to the increasing concentration of CO_2_ in the atmosphere, ocean acidification is an emerging problem. According to the published data, the global average surface ocean pH decreased by ~0.1 unit from the preindustrial era of 1770 to 2000, and it is expected that the global average annual pH of the ocean will decrease by an additional ~0.3, with buffer capacity decreasing by an average of ~34% from 2000 to 2100 (67). It is considered that, initially, with increasing pCO_2_ values in the atmosphere, the ocean will sequester more carbon by physical and biological carbon pumps. However, with subsequent decreases in buffering capacity, this tendency will decline with a change in ocean chemistry (67). The microbial community of coastal marine environments plays an important role in nutrient cycling, shoreline pollutant degradation, oxygen generation, greenhouse gas emission, ocean primary productivity, ecosystem functionality, and carbon fixation (69). Ocean acidification can lead to losses of specific components of marine microbial diversity and related ecosystem functionality, but few studies are available on these aspects. Taking the reference from the impact of nominal acidification on microbial life, we can conclude that the pH change of another ecosystem can hamper ecosystem services and diversity.

Similar to the ocean, soils are also buffered and resist pH change. The buffering capacity of soil is largely determined by the presence of organic matter, clay minerals, and carbonate compounds. It has been reported that different amendments made in the soil change the buffering capacity of soil and make it either acidic or basic (7). At very low pH soils, buffering is generally achieved by hydrolysis and precipitation of aluminum compounds (70), while calcium carbonate plays a role at alkaline or higher pH conditions (42). While at intermediate pH, cation exchange protonation and deprotonation play a role in buffering (71). The sudden influx of chemicals, pollutants, and acid can change soil’s natural buffering capacity, impacting the structure of soil microbial diversity and related ecosystem functionality. Altered pH and buffering of soil change the soil’s microbial structure and function (45). Soil microbiome plays an essential role in soil stability, chemistry, nutrient cycling, and plant growth promotion. Like ocean acidification, soil acidification can significantly impact soil functionality and crop productivity (72). In contrast to soil acidification, increased alkalinity promotes alkaliphilic microorganisms and suppresses fungi and other microbes (73). It impacts litter degradation, humus formation, and other activities, thus making the soil less fertile. More sophisticated experiments are needed to understand how soil functionality changes across different pH ranges. Like the phenomenon of gut dysbiosis, reduced buffering and altered pH conditions of the soil lead to an altered microbial community, which can lead to soil dysbiosis and hampers soil health. Microbes with similar functions to pH-sensitive communities but survival ability in a wide pH range without losing functionality could be good bioaugmentation agents or plant probiotics for soil restoration and resilience.

In addition, most of the cultivation experiments from marine samples, including sediment, water, thermal vents, and other materials, have been conducted on Zobell marine agar, seawater analogue or another common laboratory medium while neglecting the bicarbonate buffer system of the ocean. During the cultivation and physiological experiment, most of the time microbiologists do not care about buffer and use any buffer just to control the pH without knowing their cellular compatibility. The ocean is one of the least explored ecosystems in terms of microbial diversity. Neglecting the impact of media’s buffer conditions could negatively impact the enrichment and isolation of marine microbial enrichment and exploring the microbial diversity. Studies on microbial marine diversity are mainly focused on culture-independent metagenomics, which are related to community structure and neglect functional or metabolic aspects (74). The use of natural buffer conditions, unbuffered medium, or logical usage of buffer can promote the cultivation of taxa from the marine ecosystems.

The soil is a highly explored ecosystem in terms of microbial diversity cultivation and characterization. Recent data on soil metagenome indicate that despite the revival of microbial culturomics, the majority of taxa are still not covered or cultivated (75). Altered pH conditions and use of artificial buffer or non-compatible buffer systems could be one reason. In addition, most physiological studies related to soil functionality, metabolite secretion and utilisation, and the study of cross-feeding behaviors (sharing metabolites as energy and nutrient source in different microorganisms) in natural soil are generally done in artificial mediums, mostly using a non-compatible buffer system. Soil extract medium is a viable option for conducting the cultivation and physiological experiments related to soil to get more taxa and a realistic view of microbial functionality (76). Post-preservation recovery of isolated microorganisms is another big challenge. It has been found that only a fraction of marine microbial diversity survives and recovers using a conventional approach (77). The use of a non-compatible buffer system in a rehydration medium or revival medium could be one reason for suppressing the recovery of the microorganisms from preservation and dormancy, and further investigation is needed (14).

## DISCUSSION

Although the manuscript is mainly oriented towards the impact of buffers on taxonomic characterization, physiology, and cultivation of prokaryotes (bacteria and archaea), the aim of keeping the example of eukaryotes here is to present that buffer environment impacts the physiological functions and growth of both kinds of cells. It showed a broader impact of the discussed concept. In addition, the temperature of the medium impacts the buffering strength, and it could include technical inaccuracy in pH measurement during the cultivation and growth of thermo- and psychrophiles. It is found that the change in the pH value of the medium is proportional to the temperature (78). Still, this change is not substantial, and the buffering capacity of the selected compound should be explored further. Similarly, a study by Musin et al. (79) on the impact of temperature and ionic strength of medium on the buffer capacity of polyelectrolyte microcapsules reported that increasing NaCl concentration up to 1M increased the buffering strength; however, beyond that, this impact was negligible (79). Another important observation is that, due to the heavy load of organic compounds in the usual laboratory medium, titrating the pH of a rich medium with acid or base (1 N HCl and NaOH) works well with minimal change in pH. In contrast, oligotrophic and minimal mediums are prone to quick pH change due to low organic load (33). In this condition, frequent monitoring of the medium for pH stability and use of organic buffers with appropriate pH ranges which exert neutral effects on the cellular growth and physiology of selected organisms is recommended. If the buffer affects growth measurements during compatibility tests, researchers should test multiple buffers for the same pH range during pH experiments and select/recommend a more appropriate one for future research with selected organisms.

In the whole manuscript, we tried to discuss that using some buffers, mostly inorganic, sometimes becomes inhibitory and lethal for certain groups of organisms. The inhibitory activity of the buffer(s) depends on the physiological nature of the organism. Sometimes, it suppresses certain physiological activities of the organisms, and we gave ample proof of it based on the past studies presented in Table 1. This manuscript’s aim is not limited to taxonomical characteristics and descriptions of novel taxa. It is further extended to investigate the impact of buffer compounds on the cultivation of not-yet-cultured taxa and the physiological activities of the organisms. For example, if some buffer compounds inhibit the growth of certain microorganisms in that situation, we cannot capture them in the laboratory despite providing the best nutrient conditions in the culture medium. This fact should be known by each microbiologist involved in the cultivation of novel taxa. In addition, the data presented in Table 1 indicate that, although selected buffers showed no inhibitory or lethal effects on normal bacterial growth patterns, they suppress certain activities like phosphate solubilization or impair the secretion of valuable enzymes or expression of some important functional genes. Therefore, the study of the impact of buffer compounds on physiological activities is a must before planning sophisticated physiological experiments or their large-scale cultivation for commercial applications like use as bio fertilisers and probiotics. This is another crucial point that most researchers are unaware of. The manuscript mentions that blind use of recommended buffers is not appropriate, and judicious use is fine to avoid the inhibitory effects of buffer compounds during the cultivation of novel taxa, physiological experiments, and accurate pH range and optima information.

In their review article, data published by reference (80) also demonstrated that certain buffer compounds form complexes with metal ions and are not equally applicable to all biological systems. It is generally believed that zwitterionic N-substituted amino sulfonic acid buffers, which are commonly known as Good buffers, are considered more appropriate for biological experiments (65). They collected data on 31 commonly available buffers and their metal complex formation ability. However, this concept is also not universal, and some buffer compounds form complexes with metal ions that raise the chances of interference of buffers with biological components, which is not considered good.

## RESEARCH PERSPECTIVE

**Fig 3:**
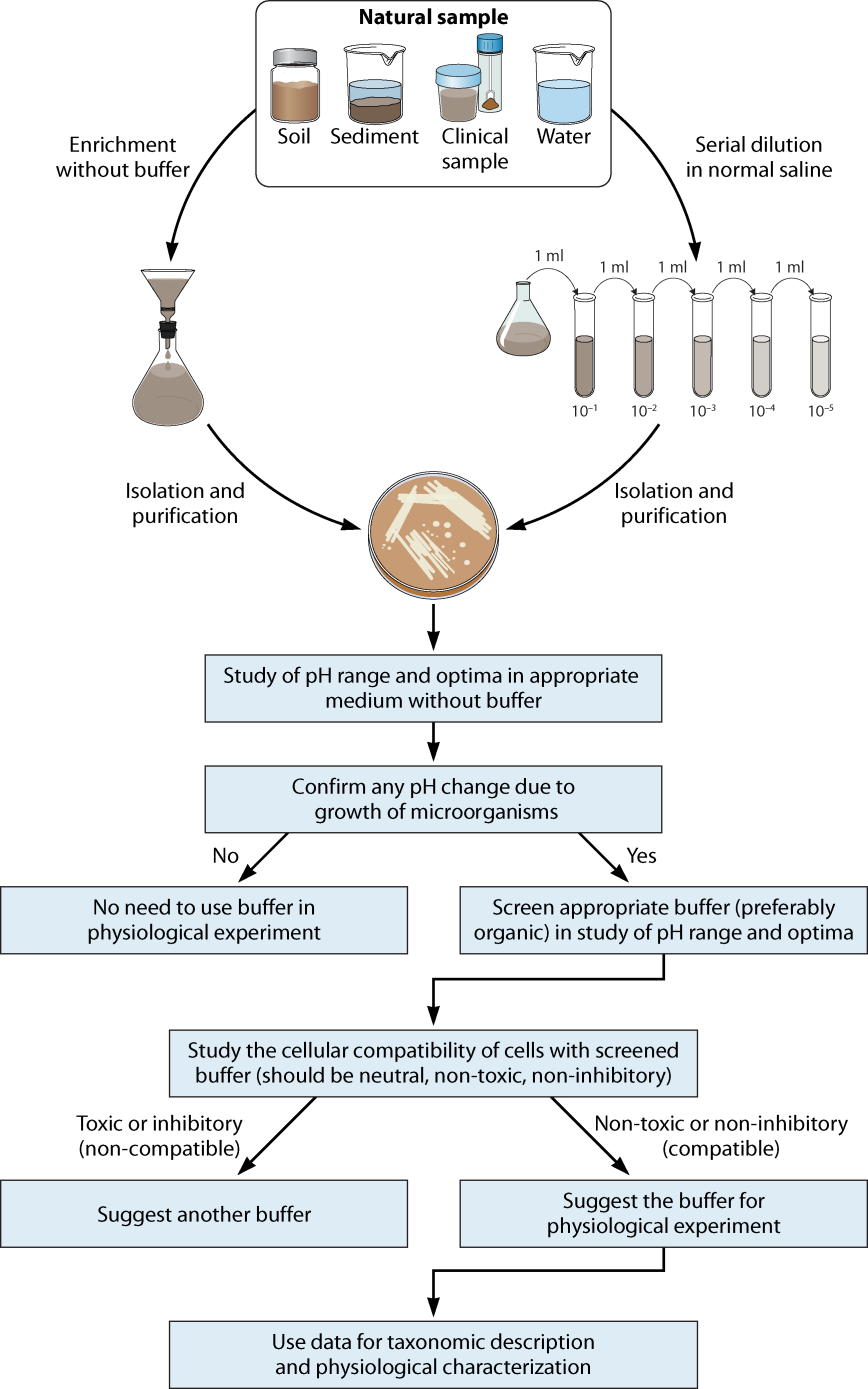
Recommendation for the selection of compatible buffer(s) for taxonomic and physiological characterization of newly isolated organisms.

A literature survey in this area indicates that researchers started thinking about the toxic and inhibitory effects of buffer compounds more than four decades ago. To date, >25 articles have been published in different areas of inhibitory effects of buffers on growth and different physiological activities of the pro- and eukaryotic cells (Tables 1 and 2). Figure 3 is a proposal to suggest future research directions based on the literature cited in this article to minimize the inhibitory effects of inorganic buffers on the cultivation and physiology of organisms. The first experimental paper on this concept was published in 1977 (43). Furthermore, a total of 16 research papers with experimentation on the impact of buffers on cellular physiology and the growth of prokaryotes have been published from 1977 to 2024 (Table 1). In addition, eight research papers have also been published on the impact of buffer compounds on eukaryotic systems from 1982 until the conceptualization of this article (Table 2). Thus, a total of 25 original experiments-based research papers have been published, reviewed, and cited in this manuscript to support the content and claims mentioned at different places in this article. In our laboratory, we realized the inhibitory effects of buffer compounds on two different *Rhodanobacter* strains during growth and physiological studies (21). In this study, we reported that some *Rhodanobacter* strains showed little or no growth at pH −5 when HOMOPIPES (homopiperazine-*N*, *N*-bis-2-(ethane sulfonic acid)) was used as a buffer. In contrast, both the selected strains of *Rhodanobacter* grew at pH 4 and below when the medium pH was simply adjusted using 1 N HCl. This finding indicates that the addition of buffers in synthetic groundwater medium exerts stress and inhibits the growth of organisms. In addition, during one of our experiments, I observed that an enrichment medium with short-chain fatty acids as substrate enriches more novel organisms than the usual enrichment medium (81, 82). Furthermore, considering the importance of the field (80), reviewed the chemistry of buffers and published a highly cited article in 2015 on a similar concept. The concept of buffer toxicity is very valuable from a diversity, ecology, and physiological point of view. The field is not young but neglected and needs more attention. Careful analysis of protocols reflects that the majority of microbiologists do not even care about the use of organic, inorganic, and biological buffers during cultivation and physiological experiments and just think about obtaining the right buffering of the medium. We believe that this article will assist in shedding light on these neglected aspects, attract the attention of microbiologists, and divert them to rethink about the judicious use of buffer compounds that are least toxic for cultivation and physiological experiments.

## CONCLUSION

In conclusion, studies and data on buffer compatibility indicate that buffers are not always safe. They can inhibit growth and adversely affect certain important physiological, biotechnological, or clinical traits of organisms. Therefore, testing the compatibility of appropriate buffer(s) before physiological experiments is a must, and if toxic or inhibitory, try to avoid that. Therefore, careful selection of buffers is mandatory in physiological experiments. Furthermore, data from microbial culturomics revealed that despite all the efforts and development in modern culturomics tools, microbial cultivability is still a concern. To date, we are only able to cultivate 1–10% of bacterial cells from different ecosystems. Most of the media used for cultivating and enriching microbes from the biological samples contains a buffer that can inhibit the growth of sensitive organisms but promotes the growth of only buffer-compatible microbiota and could be one of the factors contributing to the low cultivability of microorganisms. To boost the recovery of organisms for biodiversity and bioprospection studies, we recommend avoiding any buffer compounds during the first-time isolation of organisms from the natural environment. The goal of the microbial biodiversity study is to boost the recovery of organisms; in that context, it is advisable here that researchers use two sets of plates for the first-time isolation, one with a buffered medium and the other with an unbuffered medium. It is found that, while a single colony’s growth affects only the immediate vicinity’s pH, if the plated sample shows a high microbe count, the overall pH will change in the plate, and that will impact the growth of the slow-growing microbes. Additionally, nutrient degradation and depletion in the plate over time due to environmental factors, such as temperature or chemical instability of nutrients, will also change the pH over time. By using both types of media for isolation, microbial recovery can be improved. After isolation, further studies like optimizing the growth, studying the physiology of certain isolates, buffer compatibility, and identifying better compatible buffers need to be performed. To minimize biased growth, isolation on solid agar medium should be preferred. The possibility of change in pH and biased growth is more likely in the case of enrichment in a liquid medium due to the gradual accumulation and mixing of generated acid or alkali in the enrichment vessel. In the case of plating on solid agar medium, the biased growth in solid media is less likely because pH changes due to microbial growth and nutrient degradation are generally less impactful than in liquid cultures. This is because solid media localizes the pH changes in the colony’s vicinity.

After isolation, purification, characterization, and getting data on buffer compatibility and tolerance limit, compatible buffers should be used for physiological experiments. Even the different buffer compounds have different effects on specific physiological traits of a particular organism, and it should be optimized using different buffer systems to get unbiased data (Fig. 3).

Furthermore, a buffer compatibility test should be compulsory to avoid biased data about pH range and optima due to the effects of buffer compounds on microbial cells during taxonomical characterization. It is recommended here that after isolation and purification of novel taxa, researchers should study the pH range and optima using common laboratory media, such as nutrient broth, trypticase soy broth, R_2_A, and Luria-Bertani medium without any pH buffers with pH adjusted using 1 N HCl or 1 N NaOH except in exceptional cases wherein the organism only grows in a specific medium. However, it is also recommended that the investigator monitor the change in pH of the medium due to microbial growth during such experiments. In cases where the medium’s buffering capacity is compromised, a suitable and compatible pH buffer with only a neutral effect on cell growth must be suggested for more accurate physiological experiments with that organism in the future.
